# Nebulization of 2% lidocaine has no detectable impact on the healthy equine respiratory microbiota

**DOI:** 10.1371/journal.pone.0316079

**Published:** 2025-01-24

**Authors:** Lauren Holley, Hannah N. Creasey, Daniela Bedenice, Sarah Reed, Debora Regina Romualdo da Silva, Victoria Trautwein, Melissa Mazan, Giovanni Widmer

**Affiliations:** 1 Cummings School of Veterinary Medicine at Tufts University, Department of Clinical Sciences, North Grafton, MA, United States of America; 2 Cummings School of Veterinary Medicine at Tufts University, Department of Infectious Diseases and Global Health, North Grafton, MA, United States of America; 3 University of Connecticut, Department of Animal Science, Storrs, CT, United States of America; 4 São Paulo State University (UNESP), School of Veterinary Medicine, Araçatuba, São Paulo, Brazil; Long Island University - CW Post Campus: Long Island University, UNITED STATES OF AMERICA

## Abstract

Glucocorticosteroids remain the most common pharmaceutical approach for the treatment of equine asthma but can be associated with significant side effects, including respiratory microbiome alterations. The goal of the study was to assess the impact of 2% lidocaine nebulization, a projected alternative treatment of equine asthma, on the healthy equine respiratory microbiota. A prospective, randomized, controlled, blinded, 2-way crossover study was performed, to assess the effect of 1 mg/kg 2% lidocaine (7 treatments over 4 days) on the equine respiratory microbiota compared to control horses (saline and no treatment). Clinical assessments and respiratory samples, including nasal wash, endoscopic tracheal aspirate and bronchoalveolar lavage fluid, were obtained at each sample collection timepoint. The profile of the respiratory bacterial microbiota was evaluated using 16S amplicon sequencing, and clinical data compared using related samples analyses, based on data normality. The treatment did not affect the clinical data or alter the tracheal and nasal microbiota in healthy horses. However, time explained 12.6% of microbiota variation among samples. A significant difference in bacterial composition was observed between nasal and tracheal samples, showing the greatest relative abundance of Actinobacteria and Firmicutes, respectively. Bacterial DNA from bronchoalveolar lavage fluid did not amplify with generic primers targeting the V4 variable region of the prokaryotic small subunit ribosomal RNA gene, despite attempting multiple DNA extraction methods and PCR protocols, and after excluding PCR inhibition. This observation indicates that bronchoalveolar lavage fluid of healthy horses has a low bacterial load.

## Introduction

In contrast to the intestinal microbiota, the microbial populations in the respiratory tract have been studied less extensively, although these microorganisms are potentially important in respiratory health and disease. Based on growing evidence in humans, the respiratory microbiota shapes pathogenic processes underlying the endotypes and phenotypes of chronic respiratory conditions [1]. For example, airway hyperresponsiveness and airflow limitations in human asthma have been related to changes in bacterial populations, where increases in the relative abundance of particular bacterial species were associated with a rise in the number of inflammatory mediators [2–5]. When tracheal samples from asthmatic horses were compared to samples of healthy horses, the relative abundance of *Streptococcus* was higher and the relative abundance of *Psychrobacter*, *Rhodococcus*, *Aerococcus* and *Hymenobacter spp*. was lower in asthmatic horses. In contrast, no differences were noted between nasal samples [6]. Pulmonary bacterial communities of healthy and asthmatic horses were significantly different at the family level, but no individual bacterial family drove this difference [7].

Due to a relatively low bacterial biomass, the lung microbiota are more challenging to sequence and the risk of bacterial contamination affecting the sequence data is higher [7, 8]. Only one published study in live horses has reported 16S amplicon sequences of equine bronchoalveolar lavage fluid (BALF) samples using modified methodologies that were considered specific for ‘low-biomass’ samples [7]. This study reported significantly more bacterial DNA in nasal samples compared to BALF with an overall greater taxonomic richness and greater alpha diversity in nasal samples. In general, reported BALF bacterial communities were not dominated by any site-defining Operation Taxonomic Units (OTUs), and were composed of multiple, low-abundance OTUs [7].

The equine respiratory microbiota continues to be investigated for its role in disease pathogenesis, its ability to be manipulated for treatment purposes, and the potentially deleterious effects that certain treatments may have on its overall health. For example, equine asthma (EA) is characterized by non-septic airway inflammation and hyperresponsiveness, increased mucus secretions and chronic cough, leading to chronic poor health and performance [9]. Long-term environmental management and either systemic or inhaled administration of glucocorticosteroids is considered the mainstay of therapy. The respiratory microbiome is influenced by both environment and disease states or treatment. While environment is expected to have a dominant effect over treatment, dexamethasone nebulization was reported to decrease bacterial diversity and alter the relative abundance of eight bacterial genera in the upper respiratory tract [10]. Similarly, oral glucocorticosteroid use significantly affected the relative abundance of taxa that were enriched in asthmatic people [11]. Respiratory microbiota often differ between glucocorticosteroid-resistant and glucocorticosteroid-sensitive human asthmatic patients, where glucocorticosteroid treatment and worsening airflow has been associated with higher abundances of the genera *Lactobacillus*, *Pseudomonas* and *Rickettsia* [11]. Depletion of *Penicillium* and *Alternaria spp*. was observed in poorly controlled human asthmatics [12, 13]. Given the potentially deleterious effects of long-term glucocorticosteroid use, it is important to investigate the impact of non-steroid based asthma treatments, such as nebulized lidocaine, on respiratory microbiota.

Nebulization of lidocaine holds promise as an alternate treatment of airway inflammation in EA [14, 15]. While intravenous lidocaine had no effect on neutrophilic airway inflammation in asthmatic horses [16], nebulization of lidocaine was shown to have similar efficacy to inhaled beclomethasone and bronchodilators at suppressing cough in people, with significant improvement in clinical scoring when compared to a placebo [17, 18]. Similarly, nebulization of 1 mg/kg preservative-free lidocaine was found to be safe in asthmatic horses, reducing airway inflammation and improving clinical signs, but resulted in no change from baseline lung function testing similar to observations following nebulization with the glucocorticosteroid budesonide [14]. Assessment of the potential interaction between nebulized lidocaine and the equine respiratory microbiota could allow for further development in our understanding of the safety and efficacy of non-steroid based asthma treatments. Given the paucity of data on equine respiratory microbiota, this study aimed to examine the upper and lower respiratory microbiota of healthy horses in New England over time, and evaluated the effect of nebulization of 2% lidocaine versus isotonic saline on respiratory bacterial microbiota.

## Materials and methods

### Animal care statement

The experimental protocol was approved by the Institutional Animal Care and Use Committee of Cummings School of Veterinary Medicine at Tufts University and the University of Connecticut (IACUC protocol number: G2022-45 approved 5/2022 for Tufts Cummings School and protocol number A22-025, approved 6/2022 for The University of Connecticut).

### Animals and study design

The study used a randomized, controlled, double-blinded 2-way crossover design. Healthy non-asthmatic horses from the University of Connecticut (n = 16) were enrolled. These horses had no clinical signs or history of exercise intolerance or respiratory disorders. They were given a comparable and stable diet, were housed in an identical environment, including turn-out and access to pasture, and received the same disease prophylaxis consisting of vaccination and deworming. Horses were maintained on their regular diet for the duration of the study with free access to water. Exclusion criteria included a history of fever within the past 6 weeks, an abnormal physical examination or a serum amyloid A, a marker of acute inflammation, of > 200 ug/mL at the time of enrollment. One horse that received anti-inflammatory medication during the study period was excluded.

**Fig 1:**
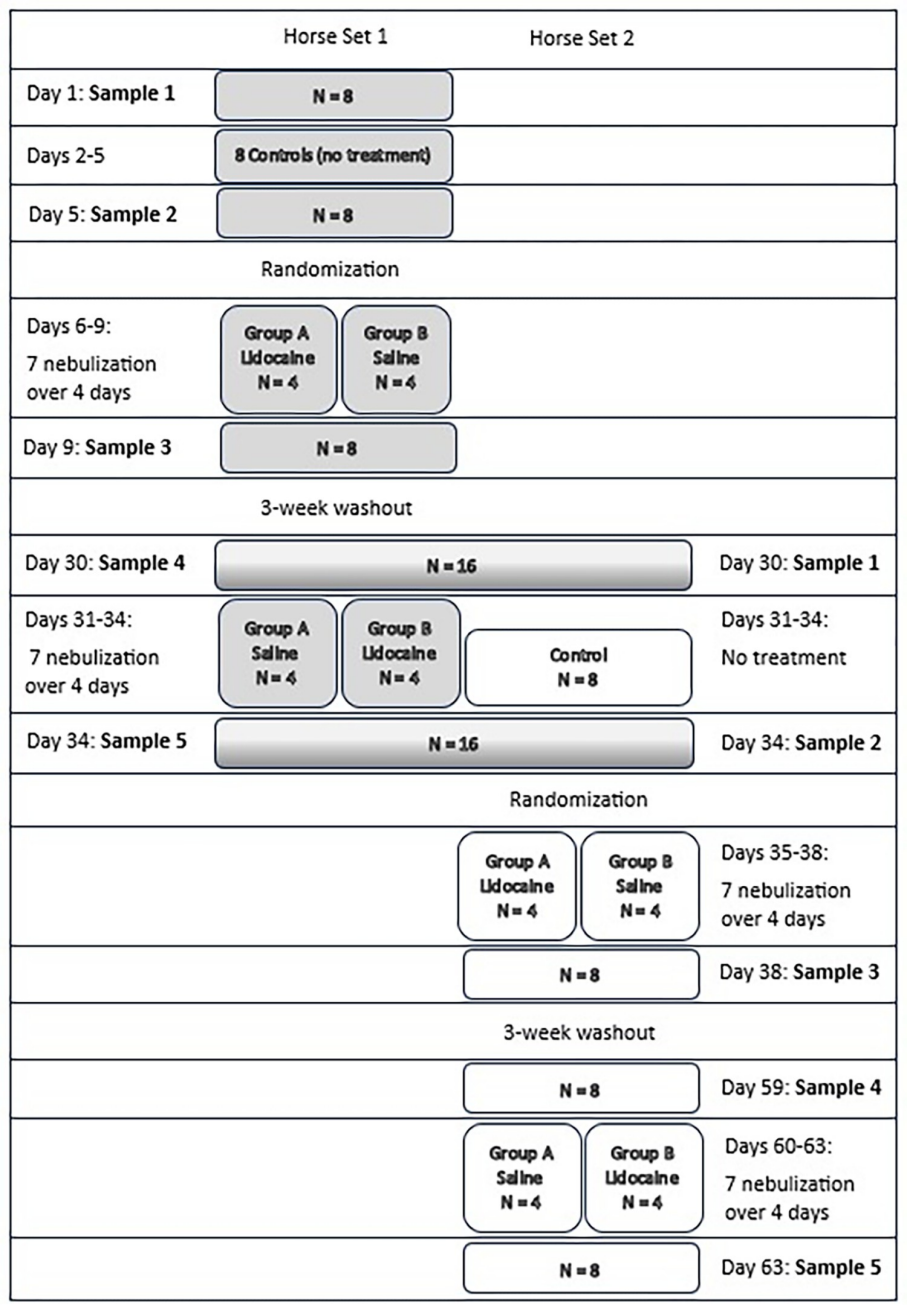
Study diagram. Grey and white colored boxes separate the two sets of horses (n = 8) that underwent the study at different time periods, while the grey/white shaded boxes indicate overlap of sample collection dates.

The study design is outlined in Fig 1. To facilitate timely sample collections, the 16 horses were divided into two groups (set 1 and 2) with 8 horses per group treated and sampled concurrently (highlighted in Fig 1). The 8 subjects in each group were further subdivided into treatment groups (A and B with 4 horses per treatment). Following initial sampling and a 4-day period without treatment between S1 and S2, each horse was randomly assigned to treatment group A or B. Group A (n = 8) was treated with 1 mg/kg of 2% preservative-containing lidocaine nebulized via Flexineb^TM^ twice daily for a total of 7 treatments over 4 days, while group B (n = 8) was nebulized twice daily for a total of 7 treatments over 4 days via Flexineb^TM^ with isotonic saline. Each horse served as its own control and treatment groups were reversed following a 3-week washout period. All horses underwent respiratory secretion sampling (nasal wash (NW), tracheal aspirate (TA) and BAL), before and after the non-treatment and treatment arm.

### Sample collection

Horses were sedated with 0.3–0.5 mg/kg intravenous xylazine hydrochloride (XylaMed Bimeda USA, Oakbrook Terrace, IL) and 0.05–0.1 mg/kg intravenous butorphanol tartrate (Torbugesic Zoetis, Parsippany, NJ). All personnel wore vinyl gloves throughout the sampling process which were changed prior to each patient and each procedure.

### Nasal wash

Both nostrils were cleaned with sterile saline and gauze to remove feed or dirt residue. A 10 Fr argyle polypropylene suction catheter (Medtronic PLC, Ireland) was advanced to the level of the medial canthus within the nares and 20 mL of sterile isotonic saline (Baxter Manufacturing, Westminster SC) was administered followed by fluid collection in a sterile cup. The process was repeated for the other nostril and collected within the same sterile collection cup.

### Tracheal aspirate

A 1.4-m videoendoscope (Olympus, Japan) was cleaned prior to each sample collection in a standardized manner. The outside portion of the endoscope was wiped with enzymatic cleaner, and 5 mL of enzymatic cleaner injected into the biopsy channel and allowed to sit for 5 min. Twenty milliliters of sterile irrigation saline (Medex Supply, Passiac NJ) were used to rinse the biopsy channel, followed by 10 mL of chlorhexiderm solution, which was allowed to sit for 5 min. The channel was then rinsed with an additional 20 mL of sterile irrigation saline, followed by 10 mL of 70% isopropyl alcohol. The outside of the scope was cleaned with 4x4 gauze and alcohol. The endoscope then sat for a further 2 min and was subsequently rinsed inside and out with sterile irrigation saline. Prior to sampling, the endoscope was flushed with sterile irrigation saline, which was processed and stored frozen on dry ice until transferred to -80°C storage.

Sterile lubricant was applied to the nostril and the endoscope was introduced through the external nares and ventral meatus into the pharynx. A volume of 10 mL of 1.6 mg/mL preservative-containing lidocaine (made with sterile isotonic saline) was applied to the pharyngeal portion of the larynx to reduce irritability and coughing. The endoscope was then advanced into the trachea to above the level of the tracheal puddle (cranial to the carina). A disposable sterile, standard type, 1.8-mm, I-CM 120-cm aspiration catheter (Endoscopy Support Services Inc., Brewster, NY) was advanced through the biopsy channel and approximately 20 cm past the end of the endoscope. A volume of 50 mL of sterile isotonic saline (Baxter Manufacturing, Westminster, SC) was instilled into the tracheal puddle and aspirated back without delay.

### Bronchoalveolar lavage

A sterile balloon-tipped BAL 300 Biovona tube (ICU Medical, San Clemente, CA) was advanced through the nares into the trachea using a standard technique, where 10 mL of 16 mg/mL preservative-containing lidocaine (made with sterile isotonic saline) were instilled into the trachea to reduce irritation and coughing. The tube was then passed until it was wedged within the lumen of a bronchus and the cuff was insufflated with air. A volume of 500 mL of sterile isotonic saline (VetOne, ID) were administered into the bronchi in 2 aliquots and sequentially aspirated using an EasyVac suction device (Precision Medical Inc., Northampton, PA). Aliquots were spun in a tabletop centrifuge at 1700 x g for 15 min. The supernatant and the pellet were recovered in 2 aliquots each. Respiratory secretion samples were immediately frozen on dry ice prior to transfer within 3 h to -80°C until DNA was extracted. BALF samples were stored on wet ice until cytological analysis.

A portable equine nebulizer Flexineb R (Flexineb^TM^ North America, Union City TN) was used for all nebulization procedures according to manufacturer’s instructions, to produce a fine particulate respirable solution. The particle size of medications given through this mask was previously validated with the appliance by Slovis et al. [Unpublished] and determined to be sufficiently small to reach the lower airways at 4.11 μm mass median diameter. A 2% preservative-containing lidocaine solution (Aspen Medical Ltd., Cleveland, OH) was administered at 1 mg/kg per nebulized dose. The nebulizer was disinfected between each horse using a standardized protocol.

### Weather data

Daily meteorological data were obtained using reported daily almanacs from the National Weather Service [19], specifying temperature ranges (in Fahrenheit), precipitation/dew points (in Fahrenheit), descriptive cloud cover and windspeeds (in mph) on sample collection dates of the current study. Meteorological descriptive data were then crudely compared to sample collection dates to determine if an effect of weather conditions was associated with microbiota differences.

### DNA extraction and 16S PCR procedures

DNA was extracted from 200 μL of sample in a QIAcube instrument using the QIAamp PowerFecal DNA kit according to manufacturer’s instructions. DNA was eluted in 50 μl elution buffer and stored at -20°C. The concentration of eluted DNA was measured with a Qbit spectrophotometer. 16S amplicon libraries were prepared essentially as described previously [20]. Briefly, the V4 variable region of the 16S ribosomal RNA gene was amplified in a 33-cycle PCR of 5 s at 95°C, 30 s 51°C and 20 s at 72°C. A 5min step at 72°C concluded the reaction. A total of 96 uniquely dual-barcoded amplicons were pooled in approximately equal proportion, as assessed using a Qbit instrument, into a sequence library. To control for technical variation, three pairs of PCR products each amplified from DNA samples extracted in parallel from the same fecal samples were included. The library was size-selected and sequenced 300-nucleotide paired-end in an Illumina MiSeq instrument operated by the Tufts University genomics core facility (tucf.org). An amplicon generated from a synthetic bacterial population (BEI Resources, cat no. HM-782D) was also included in the library as an additional quality control.

### Bronchoalveolar lavage samples

#### DNA extraction procedures

In light of the difficulties encountered when trying to PCR amplify bacterial DNA from BALF samples, the impact of the DNA extraction method on the amplification of bacterial 16S sequences was investigated. Three extraction methods were used: 1) Qiagen PowerFecal Pro (Cat. no. 51804); 2) QIAGen DNeasy Blood and Tissue Kit (Cat. no. 69516); 3) a modified version of the DNeasy method which included the following extra steps at the beginning of the DNeasy kit extraction procedure. Samples were centrifuged at 16,000 x g for 30 m, the pellets resuspended in 180 μL of enzymatic lysis buffer (20 mM Tris-HCl pH 8, 2 mM EDTA, 1.2% Triton X-100, 20 mg/mL lysozyme) and incubated for 1 h at 37°C. A volume of 200 μL AL buffer (Qiagen) supplemented with 25 μL proteinase K without ethanol was added and the samples incubated at 56°C for 30 min. Samples were then homogenized with a beadruptor (Omni, Bead Ruptor 12) for 5 min, centrifuged at 15,000 x g for 2 min, the supernatant transferred to a clean 1.5-mL microcentrifuge tube, 200 μL ethanol added and vortexed. The samples were then further processed starting at step 4 of the DNeasy kit. An additional modification included 5 freeze-thaw cycles followed by the use of N-acetyl-L-cysteine to degrade mucins. These modifications of the DNA extraction method were applied to matched aliquots of BALF pellets, BALF supernatants and unprocessed BALF samples.

#### PCR troubleshooting

In addition to various DNA extraction methods described in the preceding paragraph, several PCR protocols were tested for their ability to amplify the 16S V4 variable region of DNA extract from BALF samples. The modifications included different numbers of temperature cycles [21–24] run on two types of thermal cyclers (MJ Research PTC 100 and a MIC real-time cycler from Bio Molecular Systems). Two 16S variable regions, V1V2 and V4 [25], were targeted using several annealing temperatures. Different dilutions of the DNA template in water were also tested (undiluted, 1:1, 1:4, 1:10, and 1:100). To test if the samples contained PCR inhibitors, BALF samples were spiked with fecal DNA from a mouse [26] or *E*. *coli* DNA extracted from a laboratory strain (cat no. 25922GFP, ATCC, Manassas, Virginia, USA). Samples spiked with mouse fecal DNA were amplified with the same V4 primers as used for preparing 16S amplicons for sequencing.

#### Bioinformatics and data analysis

Program make.contigs from the *mothur* project [27] with argument trimoverlap = T was used to generate contigs from sequence pairs and eliminate non-overlapping single-stranded 3’ ends. Sequence de-noising was performed in *mothur* essentially as described [20, 28]. Operational Taxonomic Units (OTUs) were formed with a 3% sequence dissimilarity cut-off based on the OptiClust method [29]. OTUs with an average smaller than 1 sequence per sample were removed. To assess the magnitude of technical variation in the sequence data, three samples were duplicated. Duplicates were extracted individually from two portions of the same sample and each DNA sample amplified and barcoded separately. The β diversity between the three pairs of duplicated samples was 0.059, 0.091 and 0.072 weighted UniFrac distance units [30].

Sample clustering was tested using ANOSIM [31] as implemented in *mothur*. Bonferroni correction was applied for multiple comparisons. Sequences were classified against the Silva reference taxonomy (release 138.1) [32] using the Wang classifier [21]. A 75% cut-off threshold was applied. Beta-diversity between pairs of microbiota samples was quantified using weighted UniFrac [30]. To identify bacterial taxa with significantly different relative abundance in NW and TA samples, Linear Discriminant Analysis [33] was applied using program LefSe [34] as implemented in *mothur*. The phylum-level classification of OTUs with a Linear Discriminant Analysis score >2, weighed by the number of sequences in each OTU, was tabulated into a 5 x 2 weighted relative abundance vs. sample type table. The association between sample type and relative abundance was tested using a Chi-square test. Canonical Correspondence Analysis (CCA) and redundancy analysis (RDA) were used to assess the impact of one or multiple independent variables (predictors) on the microbiota. The choice of constrained ordination method depended on whether the dependent variables were best modelled by a linear or a unimodal model [35]. To test the null hypothesis of no association between dependent and independent variables, the pseudo-F statistic [22] was calculated and statistical significance determined based on 1000 iterations. CCA and RDA were performed in CANOCO, release 5.15 [22]. Shannon diversity indices were calculated using program summary.single in *mothur*.

## Results

### Clinical data

Fourteen horses were included in the final analysis; two horses were excluded from the study due to behavior and one horse was excluded from the lidocaine arm of the study due to unrelated colic requiring flunixin meglumine administration. Ten mares and 4 geldings were enrolled, with a median age of 11 (range 5–22) years. A variety of breeds were represented including 6 Thoroughbreds, 5 Morgans, 2 Quarter horses and an Andalusian. The average body condition score was 4.5/9 (range 4–6), with a mean weight of 507.3 (± 42.8) kg. All horses were part of the University of Connecticut teaching herd, with a number of different occupations including hunter-jumper (n = 7), polo (n = 4) and one of each of western pleasure, pleasure riding, and companion. Physical examination parameters and clinical respiratory score all remained within normal limits throughout the study period and no effect of treatment was identified (S1 and S2 Tables).

Meteorological review of local weather conditions determined that horse set 1 timepoints S4 and S5, and horse set 2 timepoints S1 and S2, experienced higher temperatures (89.1–91°F), higher wind speeds (24.2–29.9 mph) and maximum dew points (69–74°F) compared to horse set 2 timepoints S4 and S5 (75–77°F, 8.1–10.4 mph and 50–60°F respectively; S3 Table).

### The respiratory microbiota of New England horses

From all 53 samples combined, a total of 4.38 million V4 sequence contigs were obtained. To prevent biasing the analyses, one member of each of three replicated pairs was removed as was the synthetic control, leaving 50 samples. Sequence clustering using a 97% identity cut-off generated 19,897 OTUs. For 19,187 OTUs the average abundance per sample was <1. These rare OTUs were considered sequence noise and removed, leaving 716 OTUs in the final dataset used in the analyses.

**Fig 2:**
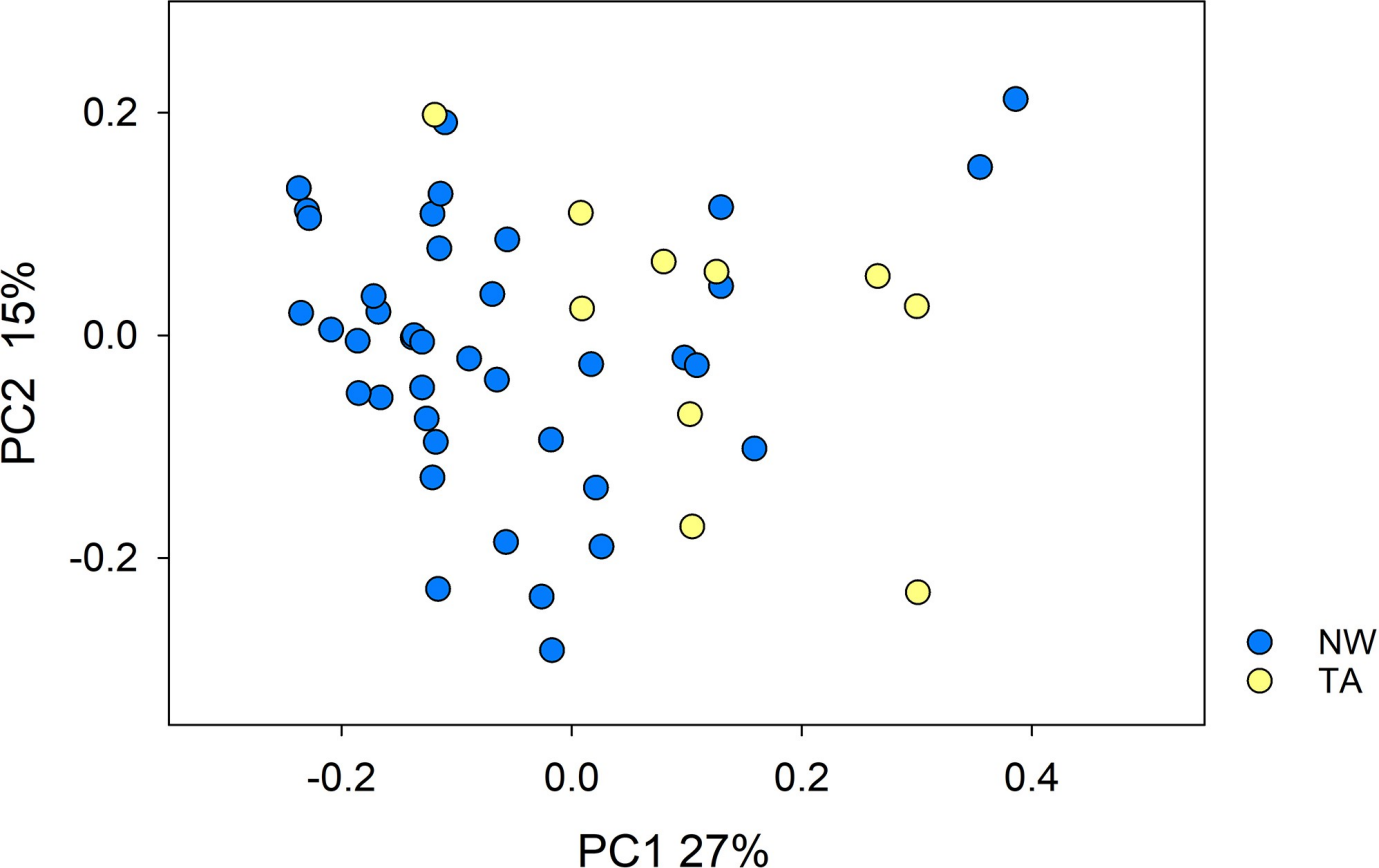
Principal coordinate analysis of 50 equine respiratory microbiota samples based on pairwise weighted UniFrac distance. Blue symbols, nasal wash (n = 40); yellow symbols, tracheal aspirate (n = 10). Clustering by sampling location is statistically significant (ANOSIM R = 0.401, p<0.0003).

The beta diversity between 50 microbiota, 40 nasal wash samples and 10 tracheal aspirates, was visualized using a principal coordinates analysis (PCoA) of weighted UniFrac distances (Fig 2). The statistical significance of the clustering by sample type was tested using ANOSIM. The test returned a significant value (R = 0.401, p<0.0003), consistent with a difference in the composition of the bacterial microbiota retrieved from the trachea and the nasal cavity. The association between sample type and OTU profile (n = 716 OTUs) was also significant according to RDA (pseudo-F = 3.3, p = 0.003). This test showed that sample type explained 6.4% of the total sequence variation. Using the Shannon α diversity metric, NW samples were slightly, but significantly, more diverse than TA samples (Mann-Whitney Rank Sum Test, p = 0.017). However, underscoring the moderate difference between the two types of samples, Berger-Parker alpha diversity was not significantly different between the two groups (p = 0.244).

**Fig 3:**
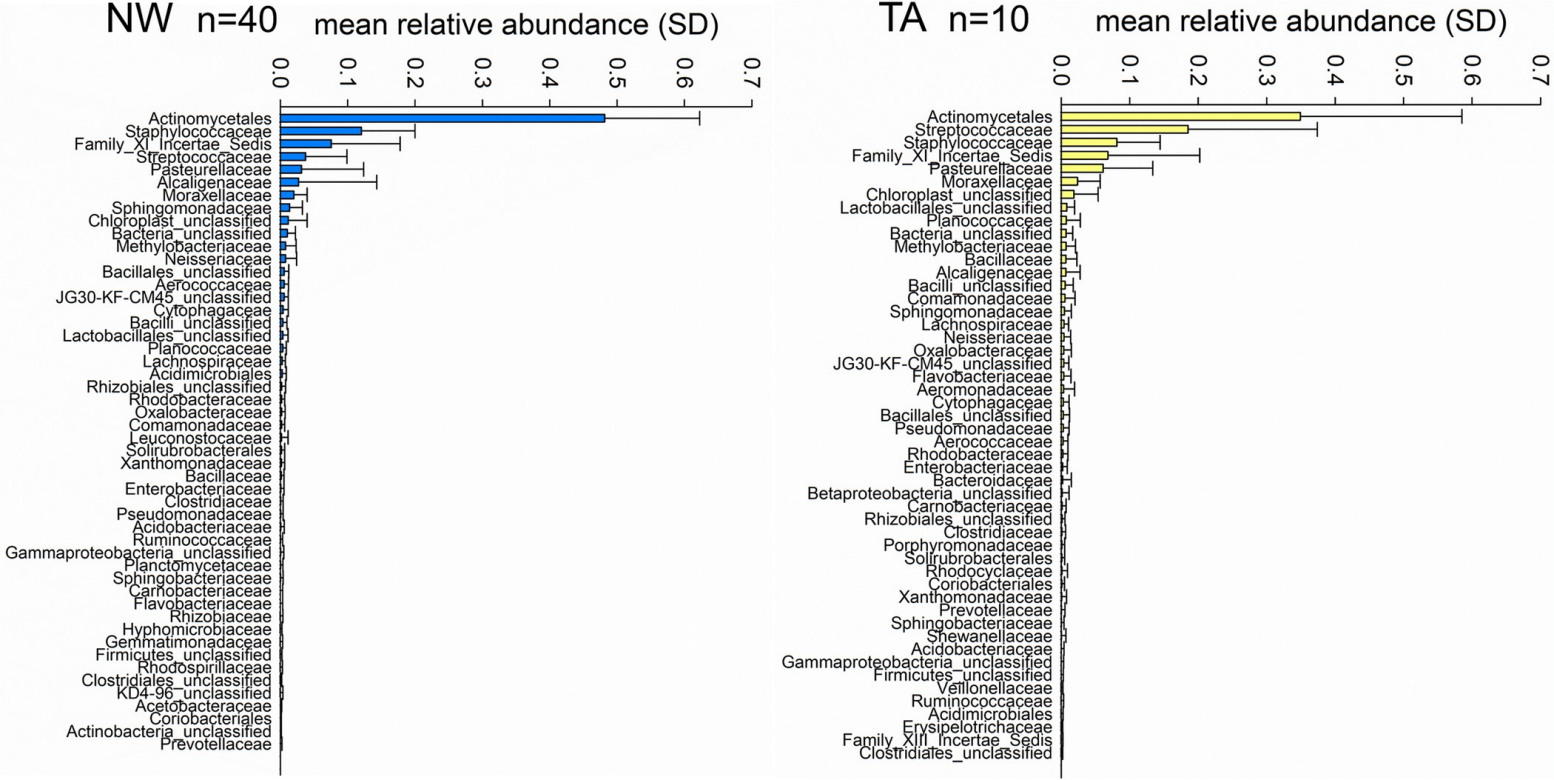
Rank-abundance plots of 50 most abundant family-level classifications in nasal wash (NW) and trachea aspirate (TA) microbiota. The relative abundance and standard deviation of each taxon is based on 40 NW and 10 TA samples. The ten most abundant NW and TA taxonomic classifications are indicated in the text. Error bars represent standard deviation.

In Fig 3 the mean relative abundance of the 50 most abundant family-level classifications out of 247 families detected in NW samples and 169 families detected in TAs are ranked from top to bottom in order of decreasing abundance. The bars show the means and standard deviation for 40 NW and 10 TA microbiota samples. The 10 most abundant NW families in order of decreasing relative abundance are Actinomycetales, Staphylococcaceae, Family_XI_Incertae_Sedis, Streptococcaceae, Pasteurellaceae, Alcaligenaceae, Moraxellaceae, Sphingomonadaceae, Chloroplast_unclassified, Bacteria_unclassified. In TA samples, Actinomycetales, Streptococcaceae, Staphylococcaceae, Family_XI_Incertae_Sedis, Pasteurellaceae, Moraxellaceae, Chloroplast_unclassified, Lactobacillales_unclassified, Planococcaceae and Bacteria_unclassified are the ten most abundant families. In contrast to previously published equine BALF and NW microbiota taxonomies [7], the rapid decrease from most abundant to rare family-level classification is consistent with the presence in natural populations of persistent, abundant species and a large number of rare and variable taxa [36].

**Fig 4:**
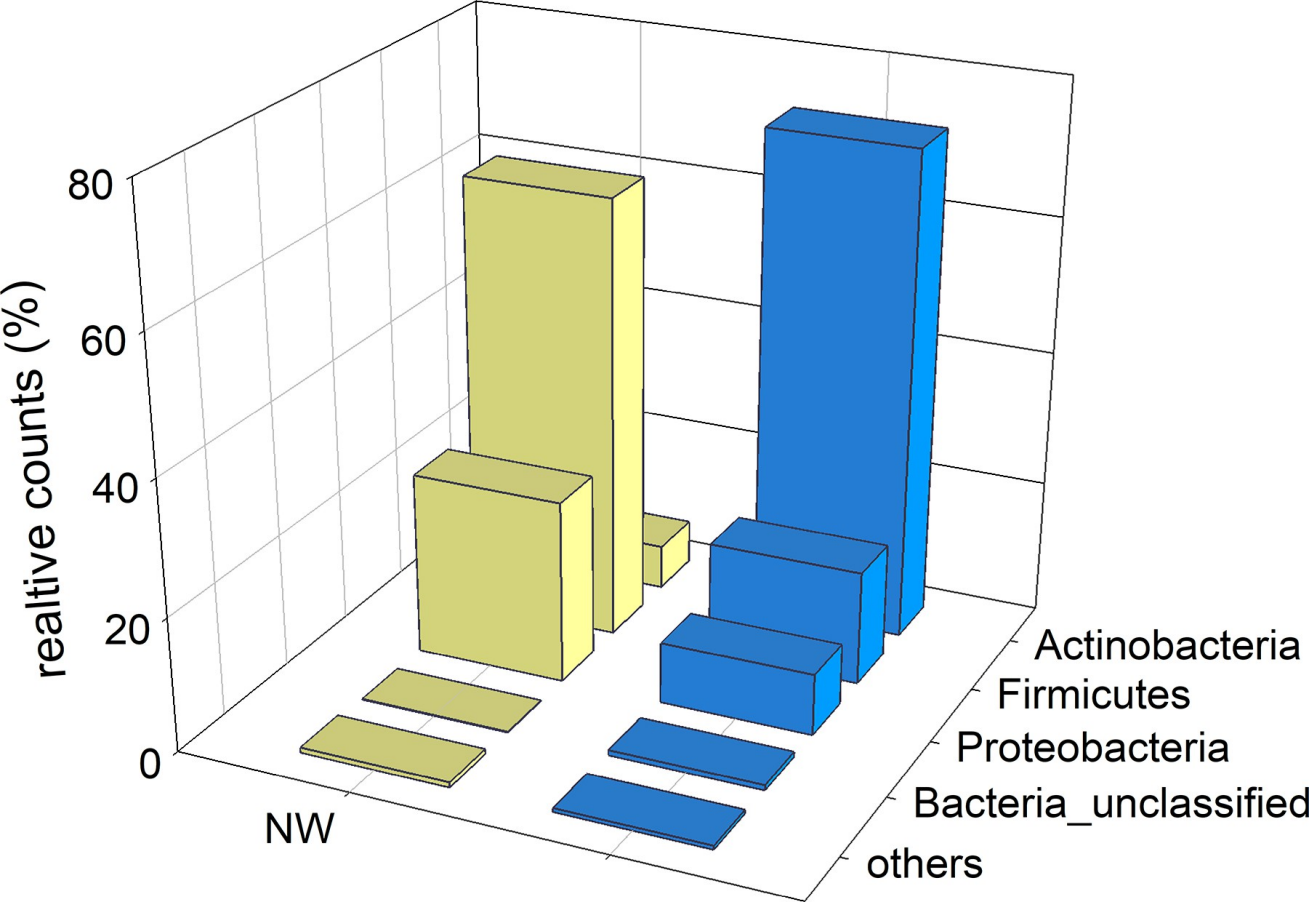
Phylum-level difference between tracheal aspirate and nasal wash microbiota identified by Linear Discriminant Analysis. The analysis is based on the classification of 4086 OTUs with a minimum of 5 sequences across 50 samples. Category “others” includes Bacteroidetes, Chloroflexi and Gemmatimonadetes.

The phylum-level taxonomy of OTUs identified by Linear Discriminant Analysis as defining the difference between NW and TA microbiota is shown in Fig 4. The graph highlights the clear difference between NW and TA microbiota, particularly with respect to the relative abundance of Actinobacteria and Firmicutes. Whereas 72% of discriminating NW sequences were classified as Actinobacteria, only 6.5% of discriminating TA sequences belonged to this taxon. In contrast, Firmicutes sequences represented 65% of discriminating TA OTUs, whereas in NW samples this phylum is much rarer, with only 17% of reads. As expected from these diverging values, the association between sample type (NW vs. TA) and abundance of defining OTUs in each phylum was statistically significant (Chi-Square = 85112, 4 d.f., p<0.001). NW-defining OTUs in the phylum Actinobacteria were taxonomically diverse, comprising Actinomycetales, Corynebacterium, Brevibacterium, Brachybacterium, Pseudonocardia, Solirubrobacter, unclassified Intrasporangiaceae, unlassified Ornithinimicrobium, unclassified Microbacteriaceae, unclassified Micrococcaceae, unclassified Micrococcineae and unclassified Propionibacteriaceae. In contrast, the TA-defining Firmicutes OTUs all belonged to the genus Streptococcus.

### Effect of lidocaine and time on NW/TA microbiota

Of particular interest was the analysis of the respiratory microbiota following lidocaine treatment. A total of 30 control samples were collected after no treatment (Fig 1, timepoints S1, S2 and S4), 10 samples were collected after saline nebulization and 10 samples after lidocaine nebulization (S3 and S5). We used RDA to test if the treatment had an effect on the OTU profile. In this analysis, treatment was defined as categorical explanatory variables with three levels (“no treatment”, saline and lidocaine) and 716 OTUs as dependent variables. Sample type (NW or TA) was defined as co-variate. This analysis returned a non-significant result (pseudo-F = 1.3, p = 0.11), indicating that, at the resolution of 16S amplicon sequencing, the type of treatment did not significantly impact the microbiota.

**Fig 5:**
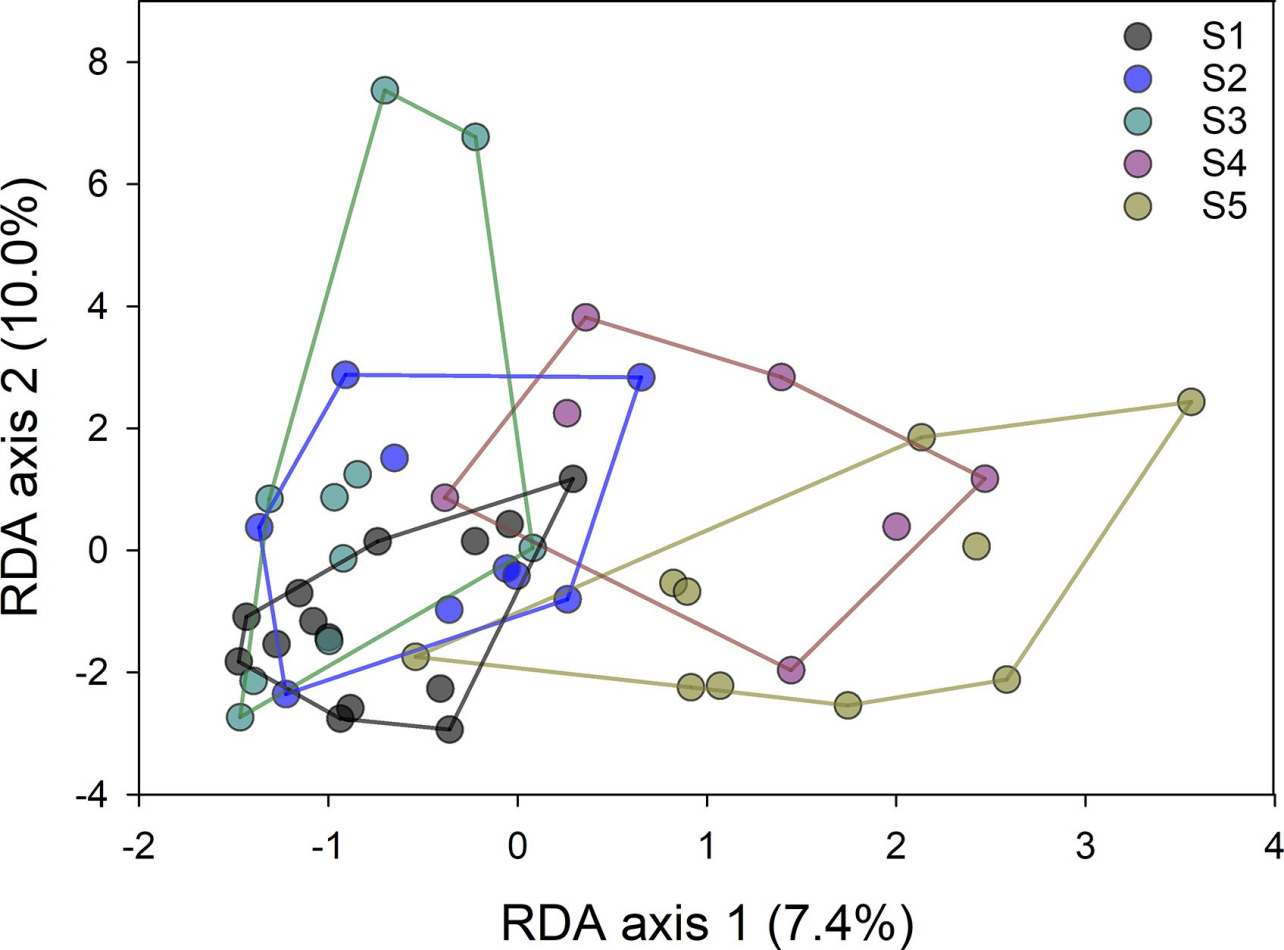
Redundancy analysis reveals temporal shift in respiratory tract microbiota. Color and envelope indicate timepoint. The analysis includes NW and TA microbiota and is based on 50 samples and 716 OTUs with a minimum average abundance of one sequence per sample.

Similarly, as for testing the effect of lidocaine nebulization described above, RDA was used to assess the effect of time on the NW and TA microbiota. Timepoints S1-S5 were input as a categorical explanatory variable with 5 levels, 716 OTUs were again input as dependent variables and the effect of sample type (NW or TA) was subtracted by defining sample type as co-variate. The test returned a significant result (pseudo-F = 1.6, p = 0.005), with time explaining 12.6% of variation. The effect of time on the microbiota is illustrated in Fig 5, where the separation between timepoints is apparent, particularly for the last two timepoints S4 and S5. Given the cross-over nature of the study, and separation of horses into two different population sets with different collection dates for S4 and S5 timepoints, no logical effect of weather was found in relation to time following evaluation of meteorological data (S3 Table). No simple explanation for this time effect was uncovered. The difference in the phylum-level OTU classification between the 30 OTUs with the best and worst fit to the variable “time” was not significant (Chi-square p = 0.21). Further, the time-dependent change in NW microbiota was not explained by a reduction in bacterial diversity that may have been caused by repeated sampling (r = 0.02, p = 0.92, n = 40). Finally, horse age was not significantly associated with microbiota profile (p = 0.31).

### Troubleshooting PCR amplification of bronchoalveolar lavage samples

DNA extracted from BALF samples consistently failed to amplify with primers targeting the V1V2 and V4 region of the bacterial 16S rRNA gene. This observation contrasted with the successful amplification of many (not all) NW and TA samples. To identify the possible causes of the consistently failing BALF DNA amplification, an extensive series of experiments was undertaken. The experiments examined the two main phases of the bench work, namely DNA extraction and PCR (Table 1). Three DNA extraction methods were used as described in Materials and Methods. The PCR conditions tested focused on different DNA concentrations, number of temperature cycles, the 16S variable region and the presence of PCR inhibitors. Given the high mucus content of BAL, we spiked samples with *E*. *coli* DNA or DNA from a synthetic bacterial population (cat. no. HM-782D, BEI Resources, Manassas, Virginia) to determine if the PCR was inhibited. Samples were also pretreated with N-acetyl-L-cysteine to degrade the mucus and assess if this treatment affected the outcome of the PCR. Based on the consistent negative outcome of these experiments, we conclude that the concentration of bacterial DNA in BALF of healthy horses is below detection limit and is not an artifact caused by inadequate DNA extraction methods or by PCR inhibition.

**Table 1:** Summary of BALF samples PCR troubleshooting.

Process	Variable	Description	Outcome
DNA extraction	extraction kit	PowerFecal DNeasy DNeasy modified	No 16S amplification detected with either method.
	mucolytic agent	N-acetyl-L-cysteine^1^	No 16S amplification detected.
PCR	number of cycles	33–35	No 16S amplification detected.
	DNA concentration	2 μL-0.02 μL^2^	No 16S amplification detected.
	annealing temperature		No 16S amplification detected.
	primers	V1V2, V4, V4 short	None of the primer pairs generated BALF amplicons.
	inhibition	spiking with *E*.*coli* DNA	No PCR inhibitors detected.

^1^ 30 mM NALC dissolved in 1.5% sodium citrate added to equal volume of sample [23]

^2^ DNA from 200 μL BALF was extracted in 50 μL water. Two μL of undiluted, 1:1, 1:4, 1:10 and 1:100 dilution were tested in the PCR.

### Lack of association between NW PCR amplification and DNA concentration

Out of 69 NW samples, 26 did not amplify any DNA with the V4 16S primers. We investigated whether the PCR outcome was related to the concentration of the extracted DNA. In this analysis, DNA concentrations below detection were set equal to 0.025 ng/μL, which is half the lowest detectable concentration reading. The mean DNA concentration of the NW samples that generated an amplicon was 0.29 ng/μL (n = 43, SD = 1.07), whereas the mean for the samples that did not amplify was 0.30 (n = 26, SD = 0.62). The difference between the groups is statistically not significant (Rank Sum test, p = 0.32). A similar conclusion was reached using a Chi2 to test the association between DNA detects and non-detects vs. PCR amplification or lack thereof (Chi2 = 1.78, 1 d.f., p = 0.18).

## Discussion

Analysis of the respiratory microbiota using 16S amplicon sequencing indicates that a multi-day 2% lidocaine treatment did not alter the nasal and tracheal microbiota of healthy horses. In contrast, intramuscular administration of dexamethasone, a glucocorticosteroid commonly used for the management of EA, was reported to affect the tracheal microbiome in both asthmatic and healthy horses, with a documented increase in eight OTUs including *Peptostreptococcus*, *Porphyromonas*, *Filifactor*, *Streptococcus*, *Parvimonas*, *Fusobacterium* and *Bacteroides spp*. and a decrease in the Candidatus_Saccharibacteria OTU of treated animals [33]. Similarly, nebulization of dexamethasone in horses with mild EA resulted in a significant decrease in nasal microbiota diversity based on Chao1 and Shannon indices, with no effect on the tracheal microbiota [37]. A significant change in the abundance of eight genera was reported, including an increase in *Alysiella*, *Bordetella¸ Acinetobacter*, *Staphylococcus*, *and Pedobacter* and a decrease *in Brevundimonas*, *Pigmentiphaga*, and an OTU assigned to the genus *Corynebacterium_1*. In contrast, lidocaine nebulization, as an alternate treatment for EA, has been shown to reduce airway inflammation and improve clinical signs in asthmatic horses [14] and resulted in no detectable microbiome alteration following nebulization in healthy horses in the current report, thus supporting its favorable safety profile [15].

Overall, corticosteroid treatment is known to influence the respiratory microbiome, likely linked with its immunomodulatory effects; however it has yet to be determined whether these influences are detrimental or beneficial. Considering that glucocorticosteroid treatment may lead to a significantly increased bronchial microbial load, including increased abundance of known pathogens, *Haemophilus* and *Moraxella* (Proteobacteria) in people, and given that glucocorticosteroids may alter the respiratory microbiota of horses, a deleterious effect is conceivable [6, 10, 38, 39]. Inhaled glucocorticosteroids, i.e., fluticasone, in conjunction with long acting β_2_ agonists, e.g., Salmeterol, have been shown to decrease α-diversity and modify the bacterial taxonomy of the microbiome in human asthmatics. Alterations in α-diversity, as an overall marker of dysbiosis, was associated with increased airway inflammation and thought to have a detrimental effect through altering immune regulation [40]. However, lone treatment with formoterol (a long acting β_2_ agonists), had no significant effects, leading to the belief that these changes may be associated with the steroid components of the combined treatment. In asthmatic horses, systemic dexamethasone only influenced the tracheal microbiome, while nebulized dexamethasone solely influenced the nasal microbiome, likely associated with the known high level of deposition in the upper airways following nebulization [6, 10, 41]. Since in the current study lidocaine nebulization did not affect the respiratory microbiome, at least not at a level detectable by 16S amplicon sequencing, and the treatment’s clinical safety has been previously established [15], nebulization of 2% lidocaine is considered safe in healthy horses. However, the effect on respiratory microbiota in asthmatic horses needs to be further investigated [14].

A previous report on horses with mild EA documented that saline nebulization significantly decreased the abundance of *Streptococcus* (Firmicutes) and *Brevundimonas* (Proteobacteria) and increased the abundance of *Pedobacter* (Bacteroidetes) in nasal samples. However, no overall difference in diversity indices in saline treated versus untreated horses was reported [37]. In the current study, neither saline nor lidocaine nebulization induced any change in microbiota in nasal and tracheal samples at the resolution of 16S amplicon sequencing. Instead, sampling time point was significantly associated with microbiota profile. However, given the randomized nature of the cross-over study, these collection time points (S4 and S5) were associated with different treatment interventions between groups. More specifically, S4 and S5 represented sampling time points pre- and post-treatment with lidocaine and saline in 7 and 7 horses, respectively. The latter observation further supports that time rather than treatment affected respiratory microbiota.

Calendar days for collection timepoints S4 and S5 differed among horses, as each treatment group was subdivided into two sets of 4 horses (subset 1 and subset 2) to facilitate effective sample collection and to ensure a cross-over treatment design (see Fig 1). For example, for the first group of treated horses, S4 and S5 time points coincided with S1 and S2 of the second group of horses, respectively. To further evaluate the influence of time on microbiota diversity, the potential impact of environment was investigated, as previous studies have highlighted the influence of environmental conditions on the respiratory microbiome [7]. Our study population was exposed to a consistent feeding/feedstuff, housing, turn-out and exercise regime, to reduce the number of uncontrolled variables as much as possible. Certain aspects of the environment, however, cannot be controlled (such as the weather) which in theory could influence the respiratory microbiome. When evaluating reported weather variables, there were distinct differences in wind and temperature between dates (S3 Table), meaning that horse set 1 (n = 7) at time points S4 and S5, were exposed to higher temperatures and windspeeds than horse set 2 at time points S4 and S5. The latter observation supports that weather was an unlikely source of the observed microbiome alterations over time (i.e., the apparent separation of microbiota at S4 and S5, Fig 5). However, repeated airway sampling and nebulization, irrespective of treatment type, may have influenced the microbiome over time. While endoscopy equipment samples were PCR-negative for bacteria, lubrication of the nose and unavoidable tissue trauma during routine procedures may influence the respiratory microbiome. By the final time points, all horses had undergone multiple nebulizations (regardless of intervention), NW, TA and BALF sample collections. No prior published reports have documented the effect of multiple BALs on respiratory microbiota, but have evaluated changes in BALF cytology and inflammatory markers following repeated airway sampling [42–44]. For example, a recent study found no clinically relevant lung inflammation in clinically healthy horses at 72 and 96 hours post-BAL, based on cytologic and cytokine evaluation [44]. It is however plausible that this semi-invasive procedure involving instillation of 500 mL sterile saline into the lower airways could alter microbial populations over time, due to changes in lower airway clearance, coughing and microtrauma affecting tracheal and nasal samples.

Previous studies investigating the respiratory microbiota of healthy equids identified Proteobacteria, Actinobacteria, Firmicutes and Bacteroidetes as the most abundant phyla [6, 7, 45]. Bray-Curtis distances between bacterial communities were reported to be significant when comparing nasal and tracheal samples of healthy horses, with Moraxella being more abundant in nasal samples and Cupriavidus more abundant in tracheal samples [6]. It was evident across multiple studies that with more distal sampling of the respiratory tract, species richness decreased [6, 10]. Microbiota within the trachea were most similar to pharyngeal microbiota, suggesting that the lower respiratory tract may be heavily influenced by the pharyngeal microbiome. *Streptococcus* was the most commonly reported bacterial genus in both the pharynx and trachea (proximal and distal) in previous reports [10, 45]. In contrast, our current study showed a significant difference in the bacterial composition of nasal and tracheal samples, which demonstrated the greatest relative abundance of Actinobacteria and Firmicutes, respectively. The latter is likely associated with external influences on the respiratory microbiota as previously established [7]. Little temporal stability among individual microbiome profiles was previously identified by others when comparing the same subject in three different environments [7]. However, greater similarities were observed between horses when samples were grouped by environment, which suggests a heavy environmental influence on the respiratory microbiome. All horses in the present study were housed in a similar and consistent environment, to reduce a confounding influence of environment on study results, thus allowing for more accurate conclusions to be made about the effect of treatment on the respiratory microbiota of horses.

The segregation of the respiratory microbiota profile according to organ (nose vs. trachea; Figs 2 and 4) was expected and is consistent with published observations [7, 8, 45, 46]. A notable difference between previous work [7] and ours, is our inability to amplify any 16S amplicons from BALF DNA. The sequence statistics reported by these authors indicate similar issues with the amplification of the 16S V4 region from BALF. Not only did several of their BALF samples fail to amplify any DNA, but sequence counts were on average more than 4 times lower than obtained from nasal and oral DNA samples, and the concentration of bacterial DNA was also lower than in other respiratory organs. The published rank-abundance plots of BALF OTUs, [7] and specifically the unusual evenness, are not typical of natural populations (microbial or other). This outcome could possibly reflect technical difficulties in obtaining 16S amplicons. The oral and nasal taxonomic profiles in the latter report, showing a small number of abundant taxa, are more typical of bacterial microbiota and were also observed in our NW and TA microbiota, as illustrated in Fig 3. In light of the results from our extensive troubleshooting experiments summarized in Table 1, we postulate that healthy horse BALF contains very low concentrations of bacteria. Horses are obligate nasal breathers, and therefore it is logical to conclude that the lower respiratory microbiota (tracheal and in theory, pulmonary) are influenced by the nasal microbiome. This is in contrast to humans, whose pulmonary and tracheal microbiome is primarily influenced by the oropharynx rather than the nasopharynx [47]. This, however, is not the only anatomical and physiologic difference between horses and many other species, including humans. The low pulmonary microbial biomass in equids in comparison to other species may be influenced by such anatomical and physiological differences. Micro-aspiration from both the oropharynx (often in the form of saliva) and gastro-esophageal reflux into the lungs has been extensively evaluated and quantified in human medicine, particularly in relation to the development of pneumonia and chronic respiratory diseases. As the adult Thoroughbred-type horse has a long trachea of approximately 75 to 80 cm, in contrast to the paltry 10–12 cm in the adult human, this greater airway length may reduce pulmonary bacterial seeding and thus reduce bacterial biomass, as supported by a low risk of community acquired pneumonia in adult horses [48]. An additional important anatomical and physiological difference between horses and humans is the essentially unidirectional flow of ingesta along the esophagus and into the stomach–indeed, regurgitation does not occur in the normal horse due to the high resting tone of the lower esophageal sphincter, the tunica muscularis conformation, and nervous system innervation that is different to most other mammals [49]. Finally, horses have long noses with extensive nasal turbinates that act as ‘scrubbers’ which prevents a majority of inhaled particulate matter from reaching the lower airways. The combination of these features likely prevents many microorganisms from reaching the lung, thus providing a potential explanation for the lack of PCR amplification of 16S V4 region in the BALF of healthy horses.

The limitations of this study relate directly to the study population and the relatively small sample size. Given the invasive, labor intensive and cost-limiting aspects of large animal research, small sample sizes are common in clinical prospective studies of horses. However, the cross-over study design wherein each horse served as its own control, reduced the negative impact of this limitation. Additionally, the current study evaluated the effect of 2% lidocaine nebulization on the respiratory microbiota of healthy horses. The latter approach is essential prior to the successful evaluation of this therapy in asthmatic patients, that show greater variability in bacterial airway populations, which hampers the assessment of treatment effect.

## Conclusions

Twice daily nebulization of 2% lidocaine was safe for administration in healthy horses, without evidence of adverse effects or treatment-associated alterations in the respiratory microbiota, as determined by 16S amplicon sequencing. However, later timepoints in sample collection were significantly associated with microbiota profile, leading to the possibility that serial sampling procedures including BAL, or serial nebulizations (irrespective of treatment type), may influence the respiratory microbiota. Inability to amplify the 16S V4 region from BALF indicates that healthy equine lungs have a low bacterial biomass.

## Supporting information

S1 Table Delta change in clinical parameters pre- (T0) and post- (T1) interventions. ^a^ Mean (standard deviation, SD) or ^b^ Median (interquartile range, IQR), based on data normality. The delta changes in clinical parameters pre- (T0) and post- (T1) interventions were neither clinically nor statistically different (p>0.05), based on paired-sample T-test for normally distributed data, and Related-samples Wilcoxon Signed Rank Test for non-normally distributed data.
(DOCX)

S2 Table Comparison of the delta change in clinical parameters before and after intervention (T1-T0) between treatment groups. ^a^ Mean (standard deviation, SD) or ^b^ Median (interquartile range, IQR). Normally distributed data were analyzed using Paired-sample T-test, and non-normally distributed data using Related-samples Wilcoxon Signed Rank Test.
(DOCX)

S3 Table Weather information for sample collection timepoints for the year 2022. ^a^ Denotes sample timepoints with significant microbiota separation based on Redundancy Analysis showing a significant temporal shift (Fig 5). Sample timepoints correlate with the phase of the study, while ‘N’ identifies the number of horses that received 7 nebulization treatments (lidocaine or saline) or no treatment. On days 30 and 34, samples from horses in 2 different phases of the study were collected concurrently, as specified in the study design of Fig 1 (S4, S1 and S5, S2 respectively).
(DOCX)
