# Supplementary material for: Nebulization of 2% lidocaine has no detectable impact on the healthy equine respiratory microbiota
Source: PLoS One. 2025 Jan 24;20(1):e0316079. doi: 10.1371/journal.pone.0316079 (PMC11759996; doi:10.1371/journal.pone.0316079)
Supplement: S1 Table — a Mean (standard deviation, SD) or b Median (interquartile range, IQR), based on data normality. The delta changes in clinical parameters pre- (T0) and post- (T1) interventions were neither clinically nor statistically different (p>0.05), based on paired-sample T-test for normally distributed data, and Related-samples Wilcoxon Signed Rank Test for non-normally distributed data. (DOCX) [file pone.0316079.s001.docx]

**Supporting information**

**Table S1**. Delta change in clinical parameters pre- (T0) and post- (T1) interventions.

| **Delta Change (T1-T0)** | **Control** ^a^ | **0.9% Saline** ^a^ | **2% Lidocaine** ^a^ |
| --- | --- | --- | --- |
| Clinical score  (out of 23) | 0 (0) | 0 (0) | 0 (0) |
| Heart rate (beats/minute) | -0.9 (6.7) | -1.7 (5.6) | 0 (7.3) |
| Respiratory rate (breaths/minute) | 1.6 (7.1) | -3.1 (6.5) | -4.0 (6) ^b^ |
| Temperature (°F) | 0.1 (0.7) | 0.4 (1.1) | 0 (0.8) |

^a^ Mean (standard deviation, SD) or ^b^ Median (interquartile range, IQR), based on data normality.

The delta changes in clinical parameters pre- (T0) and post- (T1) interventions were neither clinically nor statistically different (p>0.05), based on paired-sample T-test for normally distributed data, and Related-samples Wilcoxon Signed Rank Test for non-normally distributed data.
