# Supplementary material for: Nebulization of 2% lidocaine has no detectable impact on the healthy equine respiratory microbiota
Source: PLoS One. 2025 Jan 24;20(1):e0316079. doi: 10.1371/journal.pone.0316079 (PMC11759996; doi:10.1371/journal.pone.0316079)
Supplement: S2 Table — a Mean (standard deviation, SD) or b Median (interquartile range, IQR). Normally distributed data were analyzed using Paired-sample T-test, and non-normally distributed data using Related-samples Wilcoxon Signed Rank Test. (DOCX) [file pone.0316079.s002.docx]

**Supporting information**

**Table S2.** Comparison of the delta change in clinical parameters before and after intervention (T1-T0) between treatment groups.

| **Comparison** | **Clinical Parameter** | **N** | **Mean group difference** ^a^ | **P-value** |
| --- | --- | --- | --- | --- |
| Saline v. Control | Heart rate (beats/minute) | 14 | 0.9 (10.3) | 0.76 |
|  | Respiratory rate (breaths/minute) | 14 | 4.7 (11.6) | 0.15 |
|  | Temperature (°F) | 14 | -0.3 (1.5) | 0.48 |
| Lidocaine v. Control | Heart rate (beats/minute) | 13 | -0.9 (10) | 0.747 |
|  | Respiratory rate (breaths/minute) | 13 | 6 (7) ^b^ | 0.08 |
|  | Temperature (°F) | 13 | 0.1 (1.0) | 0.727 |
| Saline v. Lidocaine | Heart rate (beats/minute) | 13 | 0.4 (1.6) | 0.32 |
|  | Respiratory rate (breaths/minute) | 13 | 2 (6) ^b^ | 0.86 |
|  | Temperature (°F) | 13 | -1.5 (8.3) | 0.51 |

^a^ Mean (standard deviation, SD) or ^b^ Median (interquartile range, IQR).

Normally distributed data were analyzed using Paired-sample T-test, and non-normally distributed data using Related-samples Wilcoxon Signed Rank Test.
