# Supplementary material for: Nebulization of 2% lidocaine has no detectable impact on the healthy equine respiratory microbiota
Source: PLoS One. 2025 Jan 24;20(1):e0316079. doi: 10.1371/journal.pone.0316079 (PMC11759996; doi:10.1371/journal.pone.0316079)
Supplement: S3 Table — a Denotes sample timepoints with significant microbiota separation based on Redundancy Analysis showing a significant temporal shift (Fig 5). Sample timepoints correlate with the phase of the study, while ‘N’ identifies the number of horses that received 7 nebulization treatments (lidocaine or saline) or no treatment. On days 30 and 34, samples from horses in 2 different phases of the study were collected concurrently, as specified in the study design of Fig 1 (S4, S1 and S5, S2 respectively). (DOCX) [file pone.0316079.s003.docx]

**Supporting information**

**Table S3**. Weather information for sample collection timepoints for the year 2022.

| Study Day | Sample timepoint | N (number of horses) | Temperature high (°F) | Temperature low (°F) | Cloud cover | Day dew point (max °F) | Wind speed (max mph) |
| --- | --- | --- | --- | --- | --- | --- | --- |
| 1 | S1 | Control = 8  Lidocaine = 0  Saline = 0 | 75.9 | 62.1 | Overcast, mostly cloudy | 70 | 13.8 |
| 5 | S2 | Control = 7  Lidocaine = 0  Saline = 0 | 93.9 | 64 | Mostly cloudy | 71 | 24.2 |
| 9 | S3 | Control = 0  Lidocaine = 3  Saline = 4 | 84 | 63 | Overcast | 66 | 15 |
| 30 | S4 ^a^  S1 | Control = 14  Lidocaine = 0  Saline = 0 | 89.1 | 70 | Overcast, mostly cloudy | 74 | 29.9 |
| 34 | S5 ^a^  S2 | Control = 7  Lidocaine = 4  Saline = 3 | 91 | 70 | Clear to mostly, cloudy | 69 | 24.2 |
| 38 | S3 | Control = 0  Lidocaine = 4  Saline = 3 | 91.9 | 68 | Mostly cloudy | 69 | 19.6 |
| 59 | S4 ^a^ | Control = 7  Lidocaine = 0  Saline = 0 | 77 | 50 | Clear to mostly cloudy | 50 | 8.1 |
| 63 | S5 ^a^ | Control = 0  Lidocaine = 3  Saline = 4 | 75 | 66 | Overcast | 69 | 10.4 |

^a^ Denotes sample timepoints with significant microbiota separation based on Redundancy Analysis showing a significant temporal shift (Fig. 5)

Sample timepoints correlate with the phase of the study, while ‘N’ identifies the number of horses that received 7 nebulization treatments (lidocaine or saline) or no treatment. On days 30 and 34, samples from horses in 2 different phases of the study were collected concurrently, as specified in the study design of Fig 1 (S4, S1 and S5, S2 respectively).
